# Individual physiological and mitochondrial responses during 12 weeks of intensified exercise

**DOI:** 10.14814/phy2.14962

**Published:** 2021-07-29

**Authors:** Macsue Jacques, Shanie Landen, Javier Alvarez Romero, Xu Yan, Andrew Garnham, Danielle Hiam, Mélina Siegwald, Emma Mercier, Anne Hecksteden, Nir Eynon, Sarah Voisin

**Affiliations:** ^1^ Institute for Health and Sport (iHeS) Victoria University Melbourne Australia; ^2^ Australian Institute for Musculoskeletal Science (AIMSS) Melbourne Australia; ^3^ Rennes Agrocampus Ouest Rennes France; ^4^ Institute of Sports and Preventive Medicine Saarland University Saarbrücken Germany; ^5^ Murdoch Children’s Research Institute Melbourne Australia

**Keywords:** exercise, mitochondria, training variability, *V*O_2peak_

## Abstract

**Aim:**

Observed effects of exercise are highly variable between individuals, and subject‐by‐training interaction (i.e., individual response variability) is often not estimated. Here, we measured mitochondrial (citrate synthetase, cytochrome‐c oxidase, succinate dehydrogenase, and mitochondrial copy‐number), performance markers (*W*_peak_, lactate threshold [LT], and *V*O_2peak_), and fiber type proportions/expression (type I, type IIa, and type IIx) in multiple time points during 12‐week of high‐intensity interval training (HIIT) to investigate effects of exercise at the individual level.

**Methods:**

Sixteen young (age: 33.1 ± 9.0 years), healthy men (*V*O_2peak_ 35–60 ml/min/kg and BMI: 26.4 ± 4.2) from the Gene SMART study completed 12‐week of progressive HIIT. Performance markers and muscle biopsies were collected every 4 weeks. We used mixed‐models and bivariate growth models to quantify individual response and to estimate correlations between variables.

**Results:**

All performance markers exhibited significant (*W*_peak_ 0.56 ± 0.33 *p* = 0.003, LT 0.37 ± 0.35 *p* = 0.007, *V*O_2peak_ 3.81 ± 6.13 *p* = 0.02) increases overtime, with subject‐by‐training interaction being present (95% CI: *W*_peak_ 0.09–0.24, LT 0.06–0.18, *V*O_2peak_ 0.27–2.32). All other measurements did not exhibit significant changes. Fiber type IIa proportions at baseline was significantly associated with all physiological variables (*p* < 0.05), and citrate synthetase and cytochrome‐c oxidase levels at baseline and overtime (i.e., intercept and slope) presented significant covariance (*p* < 0.05). Finally, low correlations between performance and mitochondrial markers were observed.

**Conclusion:**

We identified a significant subject‐by‐training interaction for the performance markers. While for all other measures within‐subject variability was too large and interindividual differences in training efficacy could not be verified. Changes in measurements in response to exercise were not correlated, and such disconnection should be further investigated by future studies.

## Graphical abstract

Exercise training leads to whole body and tissue‐specific adaptations. However, the observed effects of exercise are highly variable between individuals, and subject‐by‐training interaction (i.e., individual response variability) is often not estimated. Here, we measured mitochondrial (citrate synthetase, cytochrome‐c oxidase, succinate dehydrogenase and mitochondrial copy‐number), performance markers (*W*_peak_, lactate threshold, and *V*O_2peak_) and fiber type proportions/expression (type I, type IIa, and type IIx) in multiple time points during 12‐week of high‐intensity interval training to investigate effects of exercise at the individual level, and if measurement changes in different domains covary each other as exercise goes by.

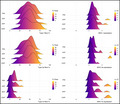

## 1 INTRODUCTION

Exercise training leads to many physiological adaptations, such as increased maximal oxygen uptake (*V*O_2max_) as well as molecular adaptations, such as mitochondrial biogenesis (Coffey & Hawley, 2007). The magnitude of these adaptations depends on the duration, intensity, volume, and type of exercise training (Hawley et al., 2014). Although the benefits of exercise are well described, large interindividual variability in the observed responses to well standardized exercise training is consistently reported (Atkinson & Batterham, 2015; Bouchard & Rankinen, 2001; Hecksteden et al., 2015; Mann et al., 2014; Timmons et al., 2005), for all exercise‐related phenotypes (Mann et al., 2014), independently of the intervention duration (Atkinson & Batterham, 2015). Furthermore, gross measures of variability in response to exercise interventions, commonly measured by pre–post approaches are not a conclusive representation of individual response variability (Hecksteden et al., 2015). Individual response (also known as subject‐by‐training interaction) relies on the assumption that consistent training changes occur for each individual (Hecksteden et al., 2015; Joyner & Lundby, 2018; Thalacker‐Mercer et al., 2013; Voisin et al., 2018). However, we and others, have shown that measuring individual response for any given variable is more complex than previously assumed, and exercise studies often fail to robustly measure it (Atkinson & Batterham, 2015; Hecksteden et al., 2015, 2018; Jacques et al., 2019; Ross et al., 2019; Voisin et al., 2018; Williamson et al., 2017).

The key to quantifying individual responses is to isolate sources of variation in exercise training responses by first quantifying the magnitude of variation in training response, given that if subject‐by‐training interaction is low assessing response of individuals is futile; and only then quantifying individual responses (Senn, 2018). In exercise studies, two stances are commonly observed as sources of variation: (1) day‐to‐day or biological variability (i.e., sleep, nutrition, etc), and (2) statistical variance such as random error. In order to isolate such sources of variation and obtain true effects of exercise training‐specific study designs and methods (Hecksteden et al., 2018; Ross et al., 2019; Voisin et al., 2018) have been proposed. With the reference standard being a replicated cross‐over design, and repeated testing measuring gradual adaptations at consecutive timepoints being a relative substitute. To date, two studies to our knowledge has implemented the repeated testing design solely for *V*O_2max_ measurement (Bonafiglia et al., 2019; Hecksteden et al., 2018), and no molecular markers have been investigated thus far.

Among the many molecular changes that are led by exercise (i.e., fiber type switch, glucose uptake, etc.), the mitochondria are known to be heavily regulated by exercise training (Bishop et al., 2014; Holloszy et al., 1970; Spina et al., 1996; Wyckelsma et al., 2017). The mitochondrion is responsible for energy production to the cells, and mitochondrial deficiency can lead to both physical and psychological disorders (Bai & Higgs, 2016; Chen & Chan, 2009; Gegg & Schapira, 2016; Schapira et al., 1990). Exercise studies often rely on isolated mitochondrial markers measures; however, one human cell contains multiple copies of mitochondria and consequently mitochondrial DNA (mtDNA). MtDNA encodes critical components of the respiratory complexes and is necessary for ATP production. An increase in mtDNA copy number (mtCN) does not necessarily equate with an increase in mitochondrial capacity and could simply be a consequence of compensatory mechanisms (i.e., reduction in mitochondrial quality and elevated mitochondrial content) (Giordano et al., 2014; Yu‐Wai‐Man et al., 2010). Thus, mitochondrial markers measured in isolation do not provide the full picture of mitochondrial health (Picard et al., 2018). Combining measures of mitochondrial content and quality is essential to access mitochondrial health. A functional index of mitochondrial health in blood has been recently proposed (Mitochondrial Health Index [MHI]), by mathematically integrating biochemical enzymatic activities and mtCN into a single score, that may represent an optimized measure of mitochondrial functional capacity (Picard et al., 2018). This method successfully captured a reduction in mitochondrial health in blood as a result of chronic psychological stress (Picard et al., 2018). However, this approach has not been explored in skeletal muscle, either in the basal state, or following a chronic physiological stimulus, such as exercise training. Furthermore, variability across mitochondrial measures is not well described, and no study to date has estimated subject‐by‐training interaction by the mitochondria.

Therefore, our aim was to use a repeated testing approach to estimate individual response for performance and molecular measures and to investigate the relationship between measurements changes overtime as a response to a 12‐week high‐intensity interval training (HIIT) intervention. We hypothesized that measuring multiple physiological and molecular components at regular intervals would allow to account for sources of variability and identification of true individual responses to exercise for performance as well as molecular measures, and by using a bivariate growth model we will also find associations between different markers in response to training.

## 2 MATERIALS AND METHODS

### 2.1 Participants

A subset of 20 participants from the Gene SMART (Skeletal Muscle Adaptive Responses to Training) study 4‐week training intervention (Yan et al., 2017) were recruited to complete a second intervention of 12 weeks. Sixteen of those 20 participants completed the full 12 weeks of HIIT (3 dropouts and 1 exclusion due to pre‐intended criterion (i.e., duplicate tests provided more than 10% difference). Nineteen completed 4 weeks (1 dropout), and 18 completed 8 weeks (1 dropout).

Participants were apparently healthy, moderately trained men (*V*O_2peak_ 35–60 ml/min/kg), aged 18–45 years old (Table S1). The study was approved by the Victoria University human ethics committee (HRE13‐223) and written informed consent was obtained from each participant. Participants were excluded from the study if they had a past history of definite or possible coronary heart disease, significant chronic or recurrent respiratory condition, significant neuromuscular, major musculoskeletal problems interfering with ability to cycle, uncontrolled endocrine and metabolic disorders or diabetes requiring insulin and other therapies (Yan et al., 2017).

### 2.2 Study design

**FIGURE 1:**
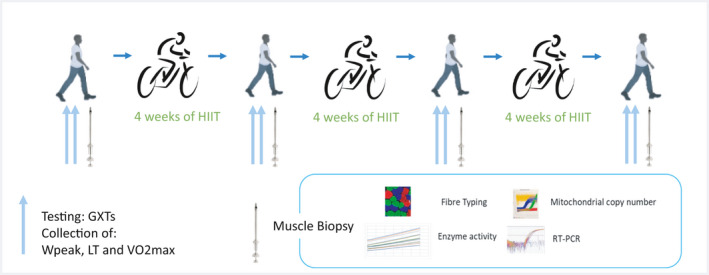
Study design. Tests and muscle biopsies were interspaced by 48 h

Participants were tested at baseline and after 4, 8, and 12 weeks of HIIT. To ensure progression, training intensity was re‐adjusted every 4 weeks based on the newly determined peak power output (*W*_peak_) and lactate threshold (LT) from the graded exhaustive exercise test (GXTs). These tests also allowed for the monitoring of individual participant progress for the longitudinal analysis of training adaptations. To increase accuracy in measurement and to reduce biological day‐to‐day variability in participants’ performance, physiological measures of fitness (*W*_peak_, LT, and *V*O_2peak_) were assessed from two GXTs, at a minimal of 2 days apart, conducted at each time point (Figure 1). Muscle biopsies were taken 48 h after performance tests from each cycle. LT was determined using the modified d‐max method (Faude et al., 2009). More details on testing criteria and participants have been described elsewhere (Yan et al., 2017).

### 2.3 Muscle biopsies

A controlled diet for 48 h prior to the muscle biopsies was provided to the participants, according to the guidelines of the Australian National Health & Medical Research Council (NHMRC). Muscle biopsies were taken using a Bergstrom needle by an experienced medical doctor from the vastus lateralis muscle of the participants’ following local anesthesia (2 ml, 1% Lidocaine [Lignocaine]). The needle was inserted in the participant leg and manual suction was applied for muscle collection. Care was taken not to contaminate the muscle samples with local anesthetic during the biopsy. Excess blood, fat, and fibrosed tissue was gently removed from biopsy if any. A piece of the muscle was immediately frozen in liquid nitrogen and stored in −80℃, and another piece was imbedded in optimum cutting temperature compound (O.C.T.), and then snap frozen and stored in −80℃ for immunology analyses. Muscle biopsies were collected at four timepoints (pre, 4W, 8W, and 12W) for comprehensive analyses of mitochondrial markers, including: citrate synthase, succinate dehydrogenase (SDH), mitochondrial copy number, cytochrome oxidase and fiber type composition (Figure 1). Due to dropouts previously described, all statistical analyses are based on 16 participants.

### 2.4 Molecular analyses and immunohistochemistry

#### 2.4.1 SDH activity (complex II activity)

We utilized the SDH activity assay kit (Colorimetric) (#ab228560) to measure maximal enzyme activity. Muscle was lysed according to the kit's protocol and 15 µl of muscle lysate was used in each reaction (well). Assay was performed in duplicates. The assay kit is able to detect less than 0.1 mU SDH activity in muscle samples. Protocol was followed according to kit user guide. SDH activities (mU/mg of tissue) were averaged and if CV >10% for the duplicate results then values were removed.

#### 2.4.2 COX activity (complex IV activity)

We utilized the assay kit (#ab239711) to measure maximal cytochrome c oxidase activity (COX). Muscle was lysed using the SDH buffer from the kit described above and 10 µl of lysate was used for each reaction (well). The activity of the enzyme was determined colorimetrically in triplicates according to the kit user guide. COX results were averaged and if CV >10% for the triplicate then divergent results were removed. COX results are presented as mol/h/kg of protein. Protein was quantified using the bicinchoninic acid assay (BCA).

#### 2.4.3 Citrate synthase activity

The most commonly used measurement of mitochondrial content is the maximal citrate synthase (CS) enzyme activity (Jacques et al., 2019; Larsen et al., 2012). Small pieces of tissue were lysed in an ice‐cold buffer (KH_2_PO_4_ & K_2_HPO_4_) using a TissueLyser II (Qiagen). Protein concentration was assessed using the BCA. Total CS activity (mol/h/kg of protein) was measured in triplicates (30℃, pH 7.5) using standard spectrophotometric assays. Values that presented a CV >10% between triplicated samples were removed.

#### 2.4.4 mtDNA copy number

Mitochondrial DNA copy number also reflects the content of mtDNA, and it is usually associated with mitochondrial gene stability and mitochondrial biogenesis (Picard et al., 2018). Mitochondrial copy number (mtCN) was determined in quadruplicates using multiplex qPCR. This method allows for simultaneous amplification of a mitochondrial (ND1) and a nuclear (RNAseP) amplicon to verify their relative abundance (Krishnan et al., 2007; Picard et al., 2018). The sequences for the ND1 amplicon (IDT) are as follows:

Forward primer (300 nM), 5’CCCTAAAACCCGCCACATCT3’; Reverse primer (300 nM): 5’GAGCGATGGTGAGAGCTAAGGT3’; and Probe (100 nM): 5’FAMCCATCACCCTCTACATCACCGCCC‐TAMRA3’. We utilized the RNAseP assay kit (Thermofisher Scientific #4403328). Taqman Universal Mastermix (Thermofisher #4304437) was used and the assay ran on a QuantStudio^™^ 7 Flex Real‐Time PCR System. The average CV for mtCN Cts was 1.02%. Data were manually curated and in cases in which samples yielded a standard deviation >0.3, the divergent sample was removed.

*Mitochondrial Health Index:* We calculated MHI as was previously reported in blood (Picard et al., 2018), using the following equation:

$$\text{MHI}=\left[\frac{\text{Energy production capacity}}{\text{Mitochondrial content}}\right]=\left[\frac{\text{Compex II}\;\left(\text{SDH}\right)+\text{Complex IV}\left(\text{COX}\right)}{\text{CS}+\text{mtDNAcn}}\right]\times 100$$

#### 2.4.5 Fiber typing (immunohistochemistry) and expression (RT‐PCR)

Exercise is known to affect fiber type composition, area, and expression (Campos et al., 2002; Taaffe & Marcus, 1997). Shifts in fiber type composition might not be achieved so easily in human interventions; however, changes in myosin heavy chain expression patterns might occur even after short interventions (Eigendorf et al., 2018). Thus, in our study, we have measured both parameters to see whether trainability is observed in either measurement.

Immunofluorescence analyses of muscle fiber types were performed on muscle tissue sections imbedded in O.C.T. Primary and secondary antibodies information have been previously described (Bloemberg & Quadrilatero, 2012). Briefly, muscle tissue was sectioned at ~8 µm, and sections were then incubated for blocking with 10% goat serum for 1 h. Primary antibodies in 10% goat serum (ThermoFisher #50062Z) (1:25) were used to incubate slides overnight in the dark at 4℃. Primary antibody was then removed, and slides washed 3 × 5 min in ddH_2_O. Secondary antibodies in 10% goat serum (1:500) were then used to incubate slides for 2 h in the dark. Slides were washed 3 × 5 min in ddH_2_O and then stained with wheat germ agglutin for 10 min in the dark (10 µg/ml). Slides were then washed once in ddH_2_O and mounted with PBS for imaging. Fiber type distribution was quantified using Fiji software and values are presented in percentage distribution. We have attempted where possible to keep our fiber counts above 150 as previously suggested (Nederveen et al., 2020), and a minimum of 100 fibers was considered the lowest threshold for analyses.

RT‐PCR for MHCs was performed on muscle tissue that was flash frozen in liquid nitrogen. RNA was extracted using the AllPrep DNA/RNA FFPE Kit (#80234 Qiagen). RNA (10 ng) was then diluted into 50 µl and reverse transcription was conducted using the iScript^™^ Reverse Transcription Supermix for RT‐qPCR (Bio‐Rad) with a thermomixer. Primers for myosin heavy chain I, IIa, and IIx used for this experiment have been described elsewhere (Eigendorf et al., 2018). RT‐PCR was conducted using the QuantumStudio‐7 (Bio‐Rad). MRNA expression levels were quantified by real‐time PCT using SYBR green fluorescence. Cycle threshold (Ct) values were normalized to a housekeeping gene, Cycl1. Samples were analyzed in triplicates and data were manually curated. In cases where samples yielded a standard deviation >0.4, the divergent sample was removed.

### 2.5 Statistical Analyses

#### 2.5.1 Responses to training at the group level and individual level (subject‐by‐training interaction)

We utilized a linear mixed model (using the lmer package in R [Kuznetsova et al., 2017]) of the form:

$$\text{Outcome}=\text{timepoint}+\text{random intercept}\;\left(\text{ID}\right)+\text{random slope}\;\left(\text{ID}\times \text{Timepoint}\right)$$

where outcome was either the physiological measure of fitness (*W*_peak_ or LT or *V*O_2peak_) or a mitochondrial marker (CS activity, mtCN, SDH, COX) or a fiber type (Type I, Type IIa, or Type IIx), timepoint was a numeric variable (0, 4, 8 or 12 weeks). The fixed effect for “timepoint” estimates whether there were changes in outcome at the group level over time (i.e., mean slope). The random intercept accounts for baseline differences in outcome between individuals, and the random slope assesses whether there are significant differences in how individuals change in outcome with time (subject‐by‐training interaction, or trainability). The individual segmental changes between the different timepoints estimates within‐subject variability.

Next, we used bivariate latent growth curve models (using the package Lavaan in R [Rosseel, 2012]) to test whether changes in physiological measurements were correlated with changes in molecular markers (i.e., correlation between slopes). A complete explanation of this method can be found in the Supplementary Files. Due to limited muscle there were missing data points therefore multiple imputations using the *mice* package was used to impute these missing values (van Buuren & Groothuis‐Oudshoorn, 2011), and results were pooled from all imputed iterations for both mixed models as well as parallel growth models with *miceadds* package (Robitzsch et al., 2021). All analyses were performed using the R software version 4.0.2.

## 3 RESULTS

### 3.1 Individual responses to 12 weeks of HIIT training are evident at the physiological level but not at the molecular level

**FIGURE 2:**
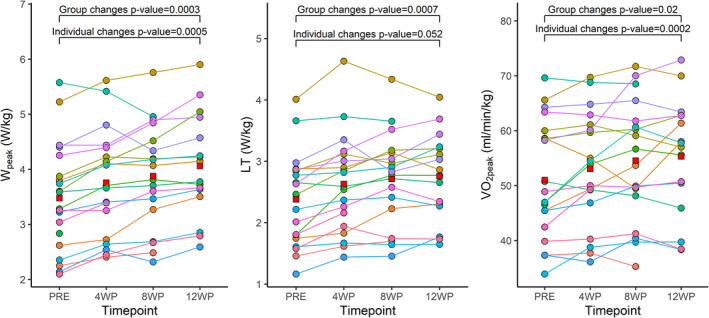
Individual changes (i.e., subject‐by‐training interaction) in physiological measures (*W*_peak_, LT, and *V*O_2peak_) after 4, 8, and 12 weeks of HIIT. Each participant is represented by a different color. Red squares represent mean values for each variable in each timepoint. HIIT, high‐intensity interval training; LT, lactate threshold

The exercise training triggered a positive physiological adaptation in a dose‐response (i.e., overtime) manner (*p* < 0.05 for all physiological variables, Table 1, Figure 2, and fixed effects Table S1).

**TABLE 1:** Group characteristics with delta changes

General info physiological variables							
	Longitudinal intervention (12 weeks)						
	Pre	4WP	8WP	12WP	Δ(4WP‐Pre)	Δ(8WP‐Pre)	Δ(12WP‐Pre)
*N*	20	19	18	16	19	18	16
Age (years)	33.07 ± 8.96	–	–	–	–	–	–
BMI (kg/m^2^)	26.40 ± 4.23	–	–	26.42 ± 3.78	–	–	–
*W*_peak_ (W/kg)	3.48 ± 0.97	3.76 ± 0.96	3.88 ± 0.95	4.06 ± 0.94	0.27 ± 0.16	0.40 ± 0.24	0.56 ± 0.33
LT (W/kg)	2.38 ± 0.74	2.63 ± 0.79	2.71 ± 0.76	2.76 ± 0.70	0.27 ± 0.22	0.33 ± 0.32	0.37 ± 0.35
*V*O_2max_ (ml/min/kg)	51.0 ± 10.6	53.1 ± 10.3	54.5 ± 11.1	55.3 ± 10.7	1.76 ± 3.04	3.36 ± 5.83	3.81 ± 6.13

Values are presented as mean ± SD.

Abbreviation: LT, lactate threshold.

**FIGURE 3:**
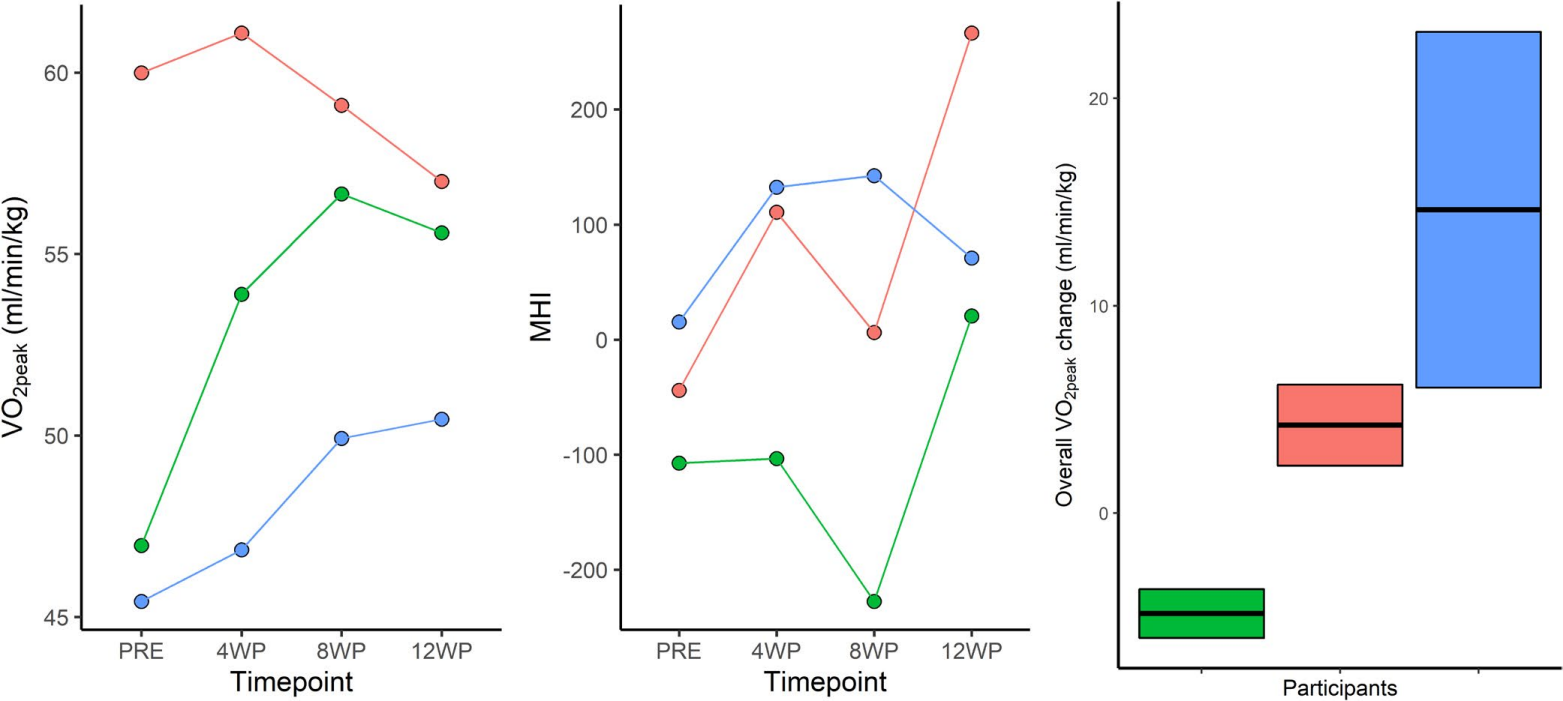
Example of subject by training interaction and comparison between whole body and molecular markers. The box plots represent individual confidence intervals for changes in *V*O_2peak_. Confidence interval: response estimate ±1.96 × SD of segmental changes

As we used a repeated testing approach, we were able to estimate within‐subject variability between multiple segments during the training period (Table S1–random effects). We separated trainability from within‐subject variability (and random error) that correspond to the error surrounding the segmental changes of the slope. We were able to delineate individual response, meaning each participant responded differently to the intervention, with some participants showing rapid and large increases in fitness, while others showed slower improvements (Figure 2). For example, in Figure 3 the highest responder presented a *V*O_2peak_ slope change of +3.34, while the lowest responder presented a slope of −2.29.

**FIGURE 4:**
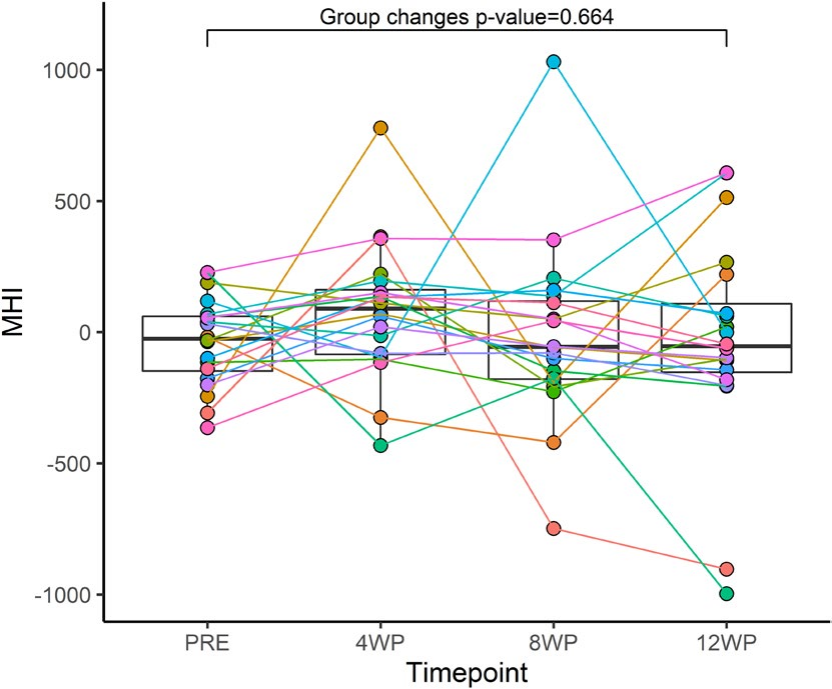
Individual changes in mitochondrial health index (MHI) for each 4‐week segment up to 12 weeks. Each participant is represented by an individual color

We did not detect any change in MHI (Figure 4) or mitochondrial markers in isolation (Figure S1) at either the group or the individual level following 12 weeks of HIIT (Table S2). Of note, mtCN was strongly associated with age in all models (*p* < 0.005) (Table S2), which is in accordance with the literature (Dolcini et al., 2020; Mengel‐From et al., 2014).

**FIGURE 5:**
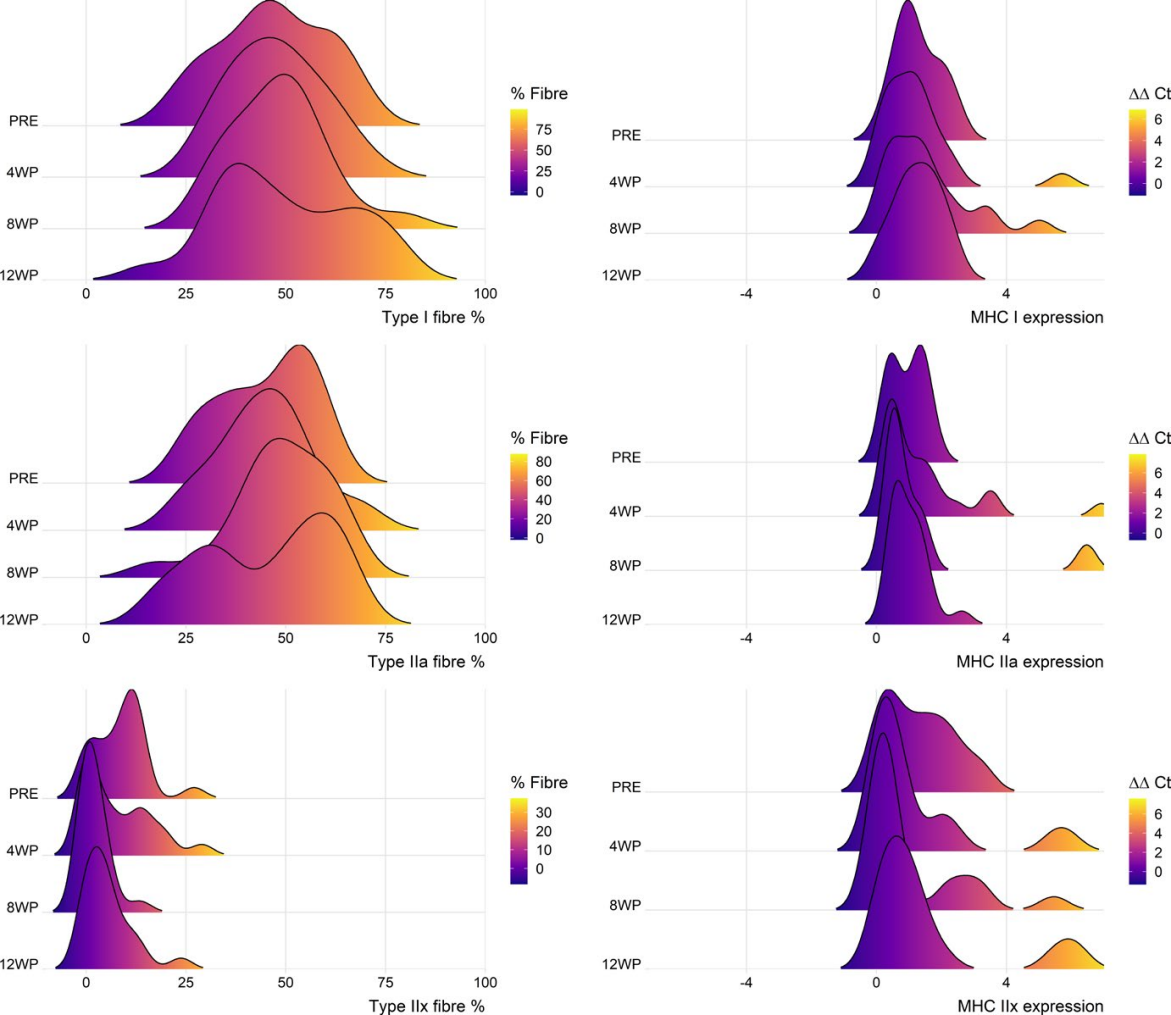
Distribution of fiber type separated by timepoint. Although minor changes are observed in the distribution overtime, those were not significant for either fiber type proportion or expression following 4, 8, and 12 weeks of HIIT. Cycle threshold (Ct). HIIT, high‐intensity interval training

Next, we investigated whether the training led to changes in fiber type distributions and whether those changes were associated with physiological or molecular changes (Tables S2 and S3). First, we tested whether fiber type proportion (as a percentage of number of fibers that were counted) and expression of myosin heavy chains (MHC) were correlated. Fiber type proportion and MHC expression presented small but significant correlation (Figure S2). We did not detect any shifts in fiber type percentage distributions or MHC expression after 12 weeks of HIIT at either the group or individual level (*p* > 0.05) (Figure 5, Table S3). However, the proportions of types I and IIa, but not IIx, were associated with physiological markers (*W*_peak_, LT and *V*O_2peak_, *p* < 0.05) (Table S3). Finally, fiber type proportion and expression were not associated with any of the mitochondrial markers after adjusting *p*‐values (*p* > 0.05, data not shown).

### 3.2 **Relationship between physiological and mitochondrial variables**–**bivariate latent growth models**

To understand the relationship between changes in molecular variables and changes in physiological variables, we built a bivariate latent growth model. Table 2 summarizes the interaction between each bivariate model. As expected, *W*_peak_, LT & *V*O_2peak_ were correlated at baseline, which means that participants with high *W*_peak_ at baseline also had high LT and *V*O_2peak_ at baseline (*p* < 0.05). A similar correlation was observed between baseline CS and COX (*p* = 0.049). *W*_peak_ and LT showed similar increases over time across participants (*p* = 0.05), but *V*O_2peak_ did not present any correlations with the other performance measurements. However, no other associations were observed between physiological and mitochondrial measures. Finally, participants with higher baseline CS displayed smaller changes in COX following training (*p* = 0.042), and vice versa, participants with higher baseline COX had smaller changes in CS following training (*p* = 0.026) (Table 2).

**TABLE 2:** Bivariate growth models: the intercept and intercept informs if variables have similar baseline values, slope and slope informs if variables have similar rate of changes, and intercept and slope informs if baseline values of one variable affects the rate of change of the other variable

Parallel growth models									
Variable	Covariates								
	Intercept & intercept			Slope & slope			Intercept & slope		
	Estimate	Std. error	*p*‐value	Estimate	Std. error	*p*‐value	Estimate	Std. error	*p*‐value
*W*_peak_ & LT	**0.574**	**0.209**	**0.006**	**0.012**	**0.006**	**0.052**	−0.009	0.039	0.826
*W*_peak_ & *V*O_2max_	**7.445**	**2.718**	**0.006**	0.06	0.063	0.344	−0.107	0.542	0.844
*W*_peak_ & CS	0.919	0.728	0.207	0.002	0.024	0.081	−0.156	0.221	0.481
*W*_peak_ & COX	0.446	0.361	0.217	−0.015	0.047	0.755	0.114	0.411	0.782
*W*_peak_ & SDH	1.956	2.218	0.378	0.133	0.14	0.344	76.867	43.992	0.081
*W*_peak_ & mtCN	0.178	0.413	0.667	−0.002	0.018	0.891	0.016	0.173	0.926
*W*_peak_ & MHI	0.416	0.293	0.156	0.026	0.037	0.484	−0.006	0.031	0.848
LT & *V*O_2max_	**6.025**	**2.256**	**0.008**	0.116	0.136	0.395	−0.245	0.498	0.624
LT & CS	0.754	0.612	0.218	0.042	0.052	0.411	−0.152	0.188	0.418
LT & COX	0.538	0.324	0.096	0.02	0.09	0.825	−0.004	0.344	0.991
LT & SDH	1.999	1.861	0.283	0.055	0.259	0.83	−0.591	0.942	−0.079
LT & mtCN	0.323	0.99	0.322	0.045	−0.786	0.434	0.196	−0.816	0.415
LT & MHI	0.227	1.162	0.245	0.008	−0.163	0.87	0.035	0.128	0.898
*V*O_2max_ & CS	3.777	8.112	0.641	0.877	0.755	0.246	−1.477	2.584	0.568
*V*O_2max_ & COX	3.354	3.831	0.381	−1.598	1.403	0.255	3.699	4.567	0.418
*V*O_2max_ & SDH	13.69	22.645	0.546	1.023	1.511	0.499	−7.738	12.254	0.528
*V*O_2max_ & mtCN	1.168	4.783	0.807	0.25	0.684	0.716	−2.101	2.946	0.491
*V*O_2max_ & MHI	2.649	2.93	0.366	−0.066	0.221	0.766	0.288	0.793	0.717
CS & COX	**2.872**	**1.461**	**0.049**	0.914	0.798	0.252	**−3.578**	**1.762**	**0.042**
CS & SDH	6.966	8.449	0.41	0.884	1.451	0.543	76.867	48.169	0.111
CS & mtCN	0.493	1.44	0.733	0.077	0.137	0.577	−0.495	0.461	0.285
COX & SDH	6.795	4.465	0.128	−1.026	2.241	0.647	−0.532	4.724	0.91
COX & mtCN	0.085	0.055	0.126	−0.126	0.095	0.188	−0.087	0.067	0.195
SDH & mtCN	−0.094	0.374	0.801	−0.006	0.072	0.933	0.285	0.219	0.194

Abbreviations: COX, cytochrome c oxidase activity; CS, citrate synthase; LT, lactate threshold; MHI, Mitochondrial Health Index; mtCN, mtDNA copy number; SDH, succinate dehydrogenase.

Significant values are highlighted in bold.

## 4 DISCUSSION

In the present study, we provided a comprehensive analysis of changes in performance, mitochondrial, and fiber type profiles of 16 young to middle aged men, at three time points throughout a 12‐week HIIT intervention. We found that performance measurements improved more consistently than molecular (mitochondrial) measurements during the 12‐week HIIT intervention. While there were clear changes in performance at the group level and we were able to establish individual response to exercise, we were unable to do so with the other markers as they were highly variable both within and between participants. Type I and IIa fibers were associated with physiological variables (*W*_peak_, LT, and *V*O_2peak_). In our growth model no significant associations were found between intercepts and slopes within same parameters, which means that baseline fitness did not influence magnitude of change for any physiological, molecular, or fiber type‐related measurement. Finally, changes at the physiological level were not associated with changes at the molecular level.

Repeated measures during exercise interventions (i.e., testing participants at multiple timepoints), constitute a cost‐effective approach to estimate individual exercise responses (Hecksteden et al., 2015, 2018; Voisin et al., 2018). Using this method, we could detect individual response for *W*_peak_, LT, and *V*O_2peak_, and identified individuals who responded better (or poorer) to the training than the group average. This methodology could therefore be the standard for studies aiming at uncovering any exercise‐related phenotype/measure at the individual level, since it has successfully isolated sources of variability providing a true trainability estimate. However, due to the lack of a control group, separating within‐subject variability and random error was not captured (Hecksteden et al., 2015, 2018).

We have assessed, for the first time in skeletal muscle, a comprehensive mitochondrial health “score”, MHI, originally assessed in blood (Picard et al., 2018). Although biologically relevant, mitochondrial markers measured in isolation are hard to interpret as compensatory mechanisms may be occurring among variables. Thus, the novel MHI measurement integrating biochemical and molecular mitochondrial measures, aims to obtain higher sensitivity to mitochondrial responses as it accounts for any relationship between variables (Picard et al., 2018). During the 12‐week HIIT intervention, the mitochondrial enzyme maximal activity and therefore the MHI were highly variable, and no consistent changes were observed at either the group or individual level. This was surprising given that mitochondrial content and function are upregulated by exercise (Holloszy et al., 1970; Spina et al., 1996; Wyckelsma et al., 2017). To ensure that the variance was not due to technical variability, we removed any duplicate results that presented a variance >10%. However, it is known that enzyme activity is highly dynamic and the timeframe in which enzymes are fired may vary both within as well as between subjects (Prouteau & Loewith, 2018). We cannot rule out the potential involvement of other enzymes in similar pathways, or enzyme Km and not the maximal activity could be different between people (Carter et al., 2001), but these hypothesis remains to be tested. Furthermore, due to the nature of skeletal muscle (i.e., multi‐nucleated) we could not account for cell number as suggested by Picard et al. The multi‐nucleated characteristic of skeletal muscle promotes the possibility that each myonuclear differ in transcriptional rates and are independently regulated and distinctive from each other, to the extent that local differences in skeletal muscle (i.e., two pieces of same biopsy) might be present following exercise (Flück et al., 2005; Islam et al., 2019; Puntschart et al., 1998), and thus potentially explaining part of the variability observed between and among measures.

Adaptive mechanisms that improve muscle function and enhance response to exercise are initiated at the transcriptional level (Egan & Zierath, 2012; Islam et al., 2019). However, apart from correlational observations there is no direct evidence linking changes in mRNA expression to training induced adaptations (Granata et al., 2018; Islam et al., 2019; Timmons et al., 2010). Here, we investigated whether fiber type gene expression was associated with fiber type composition. The proportions of fiber type have been previously reported to be associated with different modalities of exercise (Majerczak et al., 2014; Mitchell et al., 2018; Zoladz et al., 2002). For example, a high proportion of type I fibers are beneficial to endurance athletes as they are slow twitch, oxidative, and relatively more resistant to fatigue. We found significant associations between the proportions of type I and IIa fibers with physiological markers. Fiber type I was positively associated with LT and *V*O_2peak_ while fiber type IIa was negatively associated with all physiological measurements (*W*_peak_, LT, and *V*O_2peak_) (*p* < 0.05). However, neither fiber type composition nor MHC mRNA expression significantly changed with 12‐weeks of HIIT at either the group or individual level. It is recommended that for better accuracy a minimum of 150 fibers to be used for estimation of fiber type proportions (Nederveen et al., 2020), and these guidelines were followed whenever possible (i.e., large enough sample) in our study, and the absolutely minimum number of fibers considered was 100. However, even though we were careful to reduce variability within the data whenever possible, it is possible that the large noise to ratio observed by these techniques might have hindered any significant changes in our cohort (Murach et al., 2019). Further, we hypothesize that, the apparent lack of association between composition and expression may be due to the confounding influence of random error (i.e., technical error of measurement and/or biological variability) (Islam et al., 2019). A recent study investigating repeatability of exercise‐induced changes, has shown a large intra‐biopsy variation, most like to due to slight changes in the site for sampling, for fiber type distribution as well as gene expression (Islam et al., 2019), this could potentially explain the poor correlation observed between our results for fiber type composition and expression, and even the mitochondrial enzyme analysis.

Growth models allowed testing as to whether an individual who started with higher baseline values had lower improvements after training, without suffering from the statistical artefact of regression to the mean that often plagues exercise training studies (Atkinson & Batterham, 2015). To achieve this, slope factors within each system were regressed on the corresponding intercepts to control for any false associations (i.e., regression to the mean) (Wright et al., 2013). We found that baseline values did not affect the rate of change of any of the physiological or molecular variables (*p* > 0.05). Although we have conducted a power analyses for our models and our sample size is sufficient to detect with a confidence of 80% we could not derive the power of covariance between intercepts and slopes and therefore we are unsure on the certainty of this measure. New studies with repeated testing and larger sample sizes should further investigate this hypothesis. We also hypothesized that changes at the physiological level are a consequence of changes at the molecular level, and therefore physiological changes should be associated with changes molecular changes. Surprisingly, only *W*_peak_ and LT changed similarly over time, meaning that changes in most variables were independent from one another, and improvement in one variable did not necessarily mean improvement in another variable. Finally, we found an interesting relationship between CS and COX. CS and COX levels were correlated at baseline, and baseline CS values were associated with changes in COX and vice versa. CS activity is closely associated with mitochondrial content, while COX activity is strongly associated with mitochondrial oxidative phosphorylation capacity (Larsen et al., 2012; Picard et al., 2018). CS activity influences the oxidation of substrates in some respirations protocols, and complex IV is part of the mitochondrial substrate oxidation (Larsen et al., 2012), which could potentially explain the relationship observed in our results. COX/CS ratio has been previously reported to be a biochemical marker of mitochondrial dysfunction related to obesity in blood (Čapková et al., 2002). An increase of this ratio (i.e., energy‐coupled substrate oxidation) could potentially lead to increase of ATP synthesis which in turn may be channelled toward lipid formation (Katyare & Howland, 1978). Based on our results exercise might be acting as a regulator of CS/COX ratio, which might represent an important mechanism regulating adipocyte formation and reducing the risk of obesity. This is an important finding, that needs to be further explored and validated.

## 5 CONCLUSION

In summary, the repeated testing approach applied here could detect subject‐by‐training interaction for the performance markers. However, we could only estimate trainability for physiological measures of fitness, while mitochondrial markers were highly variable both between and within participants over time. We also reported a low correlation between physiological and molecular markers of fitness. Further studies utilizing the repeated testing approach in larger cohorts are needed in order to clarify the relationship between molecular and physiological responses to training. Furthermore, future studies should also include a control group for the same length in order to obtain a clear measure of random error, and then use this to normalize the effects of training. Physiological changes can also occur due to behavioral modifications that might occur during the course of the intervention (i.e., diet), and although participants were request to keep their behavior patterns, we recognize the limitation that other factors might have influenced individual response variability. Finally, variability between and within variables might due to compensatory molecular mechanisms, and other associations might be occurring such as the one reported here by CS/COX; however, further studies in the field are necessary to elucidate such networks.

## CONFLICT OF INTEREST

The authors have no conflict of interests to declare.

## AUTHORS CONTRIBUTIONS

The contribution from each author were as follows: MJ, NE, XY–conception or design of the work; MJ, SL, JA, MS, EM, SV, AG–acquisition, analysis or interpretation of data for the work; MJ, SL, JA, XY, AG, DH, MS, EM, AH, SV, NE–drafting the work or revising it critically for important intellectual content. We confirm that all authors: approved the final version of the manuscript, agreed to be accountable for all aspects of the work in ensuring that questions related to the accuracy or integrity of any part of the work are appropriately investigated and resolved, and all persons designated as authors qualify for authorship, and all those who qualify for authorship are listed.

## Supporting information



Supplementary Material
