# Supplementary material for: Individual physiological and mitochondrial responses during 12 weeks of intensified exercise
Source: Physiol Rep. 2021 Jul 29;9(15):e14962. doi: 10.14814/phy2.14962 (PMC8322753; doi:10.14814/phy2.14962)
Supplement: Supplementary file 1 — Supplementary Material [file PHY2-9-e14962-s001.docx]

**Supplementary Files**

*Individual and Parallel Growth models*. Raw means were graphed in order to verify trajectory of each variable. We estimated single and parallel Latent Growth Models (LGM) in R using the lavaan package (Rosseel, 2012). Before conduction the parallel LGMs we performed analyses in the individual variables to establish best model fit. Linear, quadratic, and piecewise models were estimated as appropriate for each variable to ensure best fit (CFI >0.95, SRMR <0.08 and chi-square p-value >0.05). A careful evaluation of the resulting parameter estimates indicates that all variances were positive and resulting parameter estimates were plausible (e.g., no Heywood cases). Once best fitting single models were estimated we progressed to conduct the parallel analyses. A figure bellow provides a conceptual diagram of the estimated models with the tracks branded as reference. The loadings for the timepoints measurements on the slope factor were fixed to 0.0 for the pre timepoint, thereby setting the start of the study as the intercept. Of most interest in this study is the covariance between the slope factors (i.e., growth rates), indicating the degree to which change in physiological and molecular markers are associated with each other (Track B in Figure 2). The estimated relationship between intercepts (Track A) captures the cross-sectional association between markers at the start of the study. The estimated growth models included a series of important covariates. First, slope factors within each system were regressed on the corresponding intercepts in order to control for any spurious association in the relationship between the growth factors (e.g., regression to the mean; Track C and D). Once this was verified slopes and intercepts were allowed to covary in order to investigate if baseline measures (intercept) affected the rate of change of participants (slope). When appropriate (i.e. better fit for the model) age was included as a covariate of the trajectories of change by regressing the latent growth factors on each. Age of entry was included to account for potentially important individual heterogeneity in development at the outset of the study because standard LGMs treat all individuals as starting at the same time. Finally, we tested whether initial status on markers had an effect on the rate of change in the other after controlling for the above-mentioned covariates (Track E and F). Individual models were required because attempts to estimate a model with growth in all variables simultaneously led to problems with specification (i.e., negative residual variances), which is not uncommon in very large structural models (Kline, 2011).


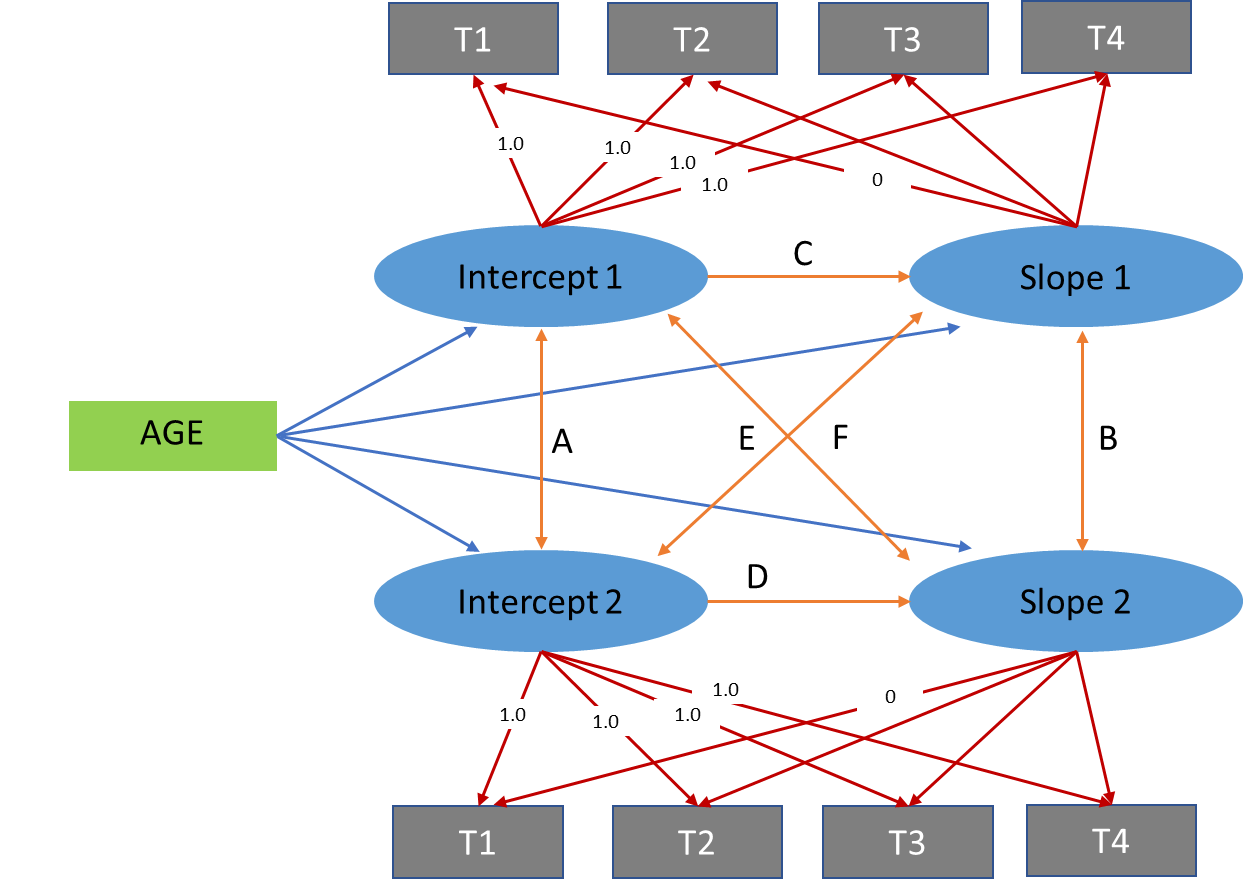


Conceptual diagram of parallel latent growth model.

Circles represent the latent variables; squares represent the manifest variables measured at each of the five timepoints. T1-T4: Timepoints Pre, 4WP, 8WP and 12WP; Single headed arrows denote regression paths, double headed arrows denote covariances; Track A: Covariance between Intercept 1 and 2; Track B: Covariance between Slope 1 and 2; Track C: Slope 1 regressed on Intercept 1; Track D: Slope 2 regressed on Intercept 2; Track E: Covariance between Slope 2 and Intercept 1; Track F: Covariance between Slope 1 and Intercept 2.


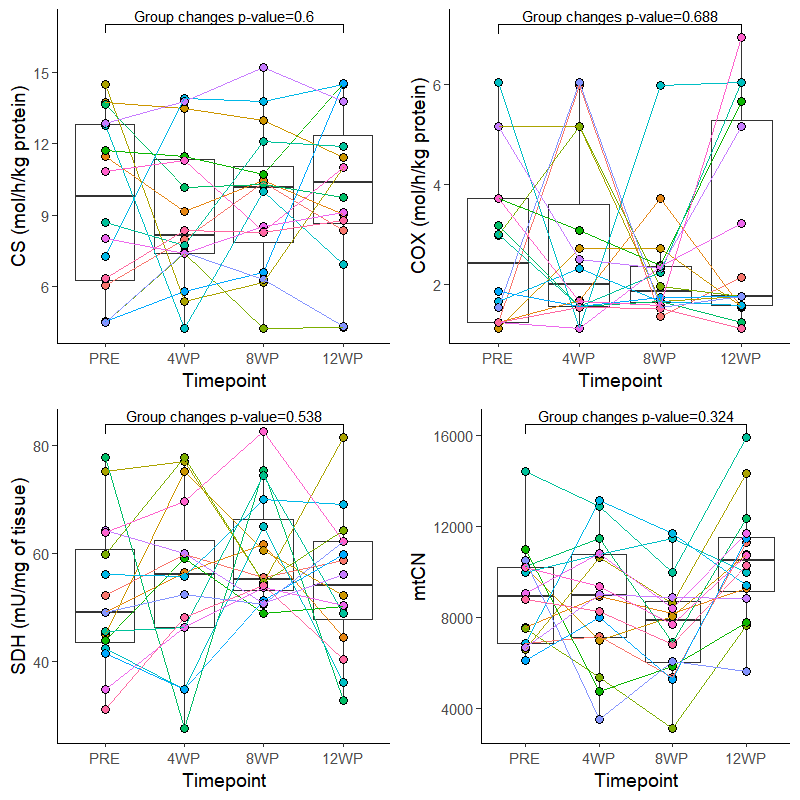


**Supplementary Figure 1:** Individual changes in Citrate Synthase (CS), the Cytochrome-C Oxidase(COX), Succinate Dehydrogenase (SDH), Mitochondrial Copy Number (mtCN), and Mitochondrial Health Index (MHI) for each 4 weeks up to 12 weeks.


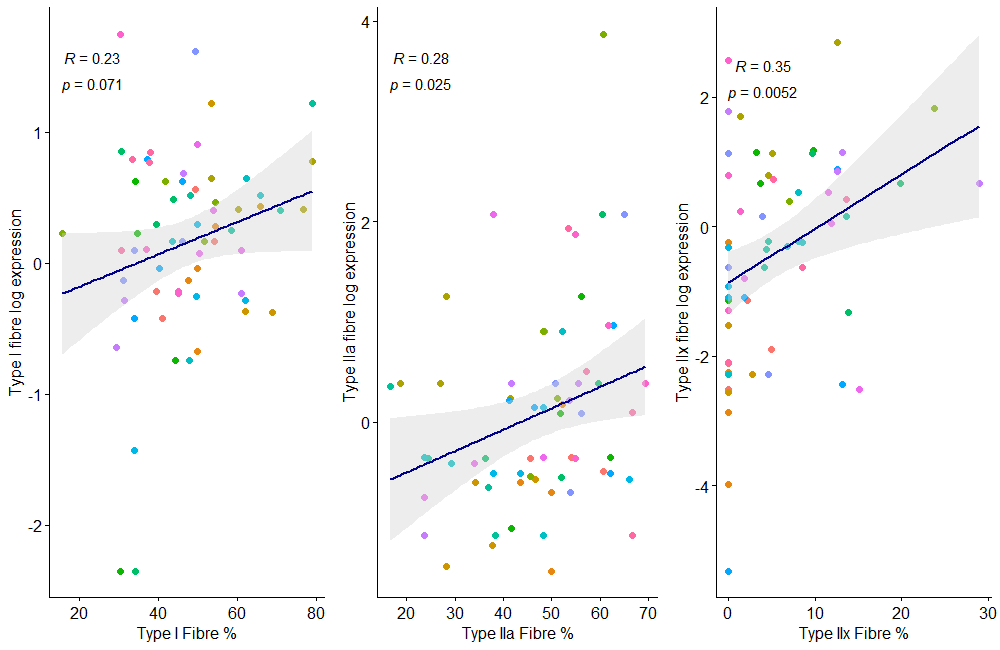


**Supplementary Figure 2**: Fibre type % and expression correlations

|  | Fixed Effects - Group Level | | | | Random Effects - Individual Level | | | | Confidence Intervals |
| --- | --- | --- | --- | --- | --- | --- | --- | --- | --- |
|  | Variable | Estimate | Standard error | p-value | Variable | Variance | Standard error | p-value | (2.5% - 97.5%) |
| W_peak_ (W/kg) | Timepoint | 0.16 | 0.04 | **0.0003** | ID x Timepoint | 0.02 | 0.14 | **0.0005** | 0.09 – 0.24 |
|  |  |  |  |  | Residual | 0.14 | 0.12 |  |  |
| LT (W/kg) | Timepoint | 0.12 | 0.03 | **0.0007** | ID x Timepoint | 0.009 | 0.09 | **0.05** | 0.06 - 0.18 |
|  |  |  |  |  | Residual | 0.027 | 0.16 |  |  |
| VO_2max_ (mL/min/kg) | Timepoint | 1.39 | 0.51 | **0.0148** | ID x Timepoint | 3.81 | 1.95 | **0.0002** | 0.27 - 2.32 |
|  |  |  |  |  | Residual | 4.54 | 2.13 |  |  |

**Supplementary Table 1:** Results from linear mixed model for physiological variables: Variable ~ Timepoint + random intercept (ID) + random slope (ID x Timepoint). Where Variable = Measurement value, Timepoint = PRE – 4WP – 8WP – 12WP. Fixed effects represent changes at the group level, while random effects represent changes at the individual level (trainability)

|  | Fixed Effects - Group Level | | | | Random Effects - Individual Level | | | |
| --- | --- | --- | --- | --- | --- | --- | --- | --- |
|  | Variable | Estimate | Standard error | p-value | Variable | Variance | Standard error | p-value |
| CS | Timepoint | 0.32 | 0.27 | 0.24 | ID x Timepoint | 0.52 | 0.45 | 0.25 |
|  | Age | 0.03 | 0.07 | 0.66 | Residual | 1.81 | 0.28 |  |
| COX | Timepoint | 0.09 | 0.19 | 0.65 | ID | 0.42 | 8.32 | 0.96 |
|  | Age | -0.002 | 0.027 | 0.93 | Residual | 1.40 | 0.19 |  |
| SDH | Timepoint | 1.62 | 1.42 | 0.25 | ID | 4.7 | 2.34 | 0.06 |
|  | Age | -0.25 | 0.22 | 0.25 | Residual | 11.54 | 1.23 |  |
| mtCN | Timepoint | 311.24 | 263.18 | 0.24 | ID | 775.16 | 535.21 | 0.15 |
|  | Age | 130.09 | 41.25 | **0.002** | Residual | 2232.52 | 248.25 |  |
| MHI | Timepoint | 21.19 | 35.67 | 0.56 | ID x Timepoint | 6671 | 81.68 | 0.096 |
|  | Age | 4.11 | 4.65 | 0.88 | Residual | 68412 | 261.56 |  |

**Supplementary Table 2**: Results from linear mixed model for mitochondrial measures. Linear mixed model: Variable ~ Timepoint + random intercept (ID) + random slope (ID x Timepoint). Where Variable = Measurement value, Timepoint = PRE – 4WP – 8WP – 12WP. Fixed effects represent changes at the group level, while random effects represent changes at the individual level (trainability)

|  | Fixed Effects - Group Level | | | | Random Effects - Individual Level | | | |
| --- | --- | --- | --- | --- | --- | --- | --- | --- |
|  | Variable | Regression coefficient | Standard error | p-value | Variable | Variance | Standard error | p-value |
| Fibre Type I % | Timepoint | -0.97 | 1.27 | 0.45 | ID x Timepoint | 2.67 | 1.64 | 0.79 |
|  | W_peak_ | 4.67 | 2.41 | 0.07 | Residual | 94.83 | 9.74 |  |
|  | Timepoint | -0.88 | 1.19 | 0.47 | ID x Timepoint | 0.91 | 0.95 | 0.83 |
|  | LT | 6.39 | 2.77 | **0.03** | Residual | 96.38 | 9.82 |  |
|  | Timepoint | -0.85 | 0.21 | 0.46 | ID x Timepoint | 0.26 | 0.51 | 0.94 |
|  | VO_2peak_ | 0.58 | 0.21 | **0.01** | Residual | 93.32 | 9.66 |  |
| Fibre Type IIa % | Timepoint | 1.86 | 1.36 | 0.18 | ID x Timepoint | 0.84 | 0.92 | 0.79 |
|  | W_peak_ | -6.03 | 1.91 | **0.005** | Residual | 127.62 | 11.29 |  |
|  | Timepoint | 1.86 | 1.17 | 0.12 | ID x Timepoint | 0.39 | 0.62 | 0.91 |
|  | LT | -5.66 | 2.71 | **0.05** | Residual | 94.51 | 9.72 |  |
|  | Timepoint | 1.81 | 1.14 | 0.12 | ID x Timepoint | 0.29 | 0.54 | 0.93 |
|  | VO_2peak_ | -0.5 | 0.19 | **0.02** | Residual | 94.02 | 9.69 |  |
| Fibre Type IIx % | Timepoint | -0.98 | 4.46 | 0.196 | ID x Timepoint | 1.01 | 0.91 | 0.27 |
|  | W_peak_ | 0.43 | 1.22 | 0.722 | Residual | 5.86 | 0.70 |  |
|  | Timepoint | -0.94 | 0.78 | 0.23 | ID x Timepoint | 0.98 | 0.92 | 0.29 |
|  | LT | -0.31 | 1.45 | 0.83 | Residual | 5.87 | 0.71 |  |
|  | Timepoint | -0.89 | 0.78 | 0.25 | ID x Timepoint | 0.97 | 0.89 | 0.27 |
|  | VO_2peak_ | -0.07 | 0.11 | 0.55 | Residual | 5.83 | 0.70 |  |
| Fibre Type I (log expression) | Timepoint | -0.005 | 0.09 | 0.95 | ID x Timepoint | 0.004 | 0.06 | 0.95 |
|  | W_peak_ | -0.005 | 0.11 | 0.96 | Residual | 0.53 | 0.73 |  |
|  | Timepoint | -0.007 | 0.08 | 0.93 | ID x Timepoint | 0.003 | 0.06 | 0.96 |
|  | LT | 0.003 | 0.14 | 0.98 | Residual | 0.53 | 0.73 |  |
|  | Timepoint | -0.01 | 0.08 | 0.87 | ID x Timepoint | 0.001 | 0.04 | 0.98 |
|  | VO_2peak_ | 0.005 | 0.01 | 0.61 | Residual | 0.53 | 0.73 |  |
| Fibre Type IIa (log expression) | Timepoint | 0.12 | 0.11 | 0.32 | ID x Timepoint | 0.02 | 0.15 | 0.89 |
|  | W_peak_ | -0.18 | 0.14 | 0.21 | Residual | 0.88 | 0.94 |  |
|  | Timepoint | 0.12 | 0.11 | 0.31 | ID x Timepoint | 0.02 | 0.14 | 0.88 |
|  | LT | -0.29 | 0.17 | 0.11 | Residual | 0.87 | 0.93 |  |
|  | Timepoint | 0.10 | 0.11 | 0.36 | ID x Timepoint | 0.01 | 0.11 | 0.97 |
|  | VO_2peak_ | -0.02 | 0.01 | 0.25 | Residual | 0.91 | 0.95 |  |
| Fibre Type IIx (log expression) | Timepoint | -0.05 | 0.16 | 0.76 | ID x Timepoint | 0.03 | 0.18 | 0.57 |
|  | W_peak_ | 0.23 | 0.03 | 0.46 | Residual | 1.55 | 1.24 |  |
|  | Timepoint | -0.02 | 0.15 | 0.90 | ID x Timepoint | 0.03 | 0.16 | 0.65 |
|  | LT | 0.09 | 0.36 | 0.79 | Residual | 1.57 | 1.25 |  |
|  | Timepoint | -0.02 | 0.15 | 0.87 | ID x Timepoint | 0.03 | 0.17 | 0.62 |
|  | VO_2peak_ | 0.01 | 0.03 | 0.63 | Residual | 1.56 | 1.25 |  |

**Supplementary Table 3**: Results from linear mixed model for fibre type measures. Linear mixed model: Variable ~ Timepoint + random intercept (ID) + random slope (ID x Timepoint). Where Variable = Measurement value, Timepoint = PRE – 4WP – 8WP – 12WP. Fixed effects represent changes at the group level, while random effects represent changes at the individual level (trainability)
